# Age as Risk Factor for Death from Pandemic (H1N1) 2009, Chile

**DOI:** 10.3201/eid1707.101398

**Published:** 2011-07

**Authors:** Jeannette Dabanch, Cecilia Perret, Manuel Nájera, Claudia González, Andrea Guerrero, Andrea Olea, Rodrigo Fasce, Cecilia Morales, Jeanette Vega, for the Advisory Committee of the Chilean Ministry of Health

**Affiliations:** Author affilations: Universidad de los Andes Hospital Militar, Santiago, Chile (J. Dabanch);; Pontificia Universidad Católica de Chile, Santiago (C. Perret);; Chilean Ministry of Health, Santiago (M. Nájera, C. González, A. Guerrero, A. Olea, C. Morales, J. Vega);; Public Health Institute of Chile, Santiago (R. Fasce)

**Keywords:** pandemic (H1N1) 2009, influenza, viruses, risk factors, age, mortality rate, elderly persons, Chile, dispatch

## Abstract

Pandemic (H1N1) 2009 affected Chile during the winter of 2009. The hospitalization rate was 0.56% overall and 3.47% for persons >60 years of age at risk for severe disease and death independent of concurrent conditions. Age >60 years was the major risk factor for death from pandemic (H1N1) 2009.

On April 23, 2009, the World Health Organization issued alerts about the emergence of pandemic (H1N1) 2009. On May 17, during fall in the Southern Hemisphere, the first case of this disease in Chile was identified (*1*). Pandemic (H1N1) 2009 then replaced seasonal influenza, which had accounted for <1% of confirmed influenza cases in Chile (*2*). Immediately after the alerts, a national mandatory notification system was started in Chile for influenza-like illness (ILI) cases and hospitalized persons with pandemic (H1N1) 2009 in public and private institutions. We analyzed data for patients with confirmed pandemic (H1N1) 2009 hospitalized during epidemiologic weeks 20–32 (May 19–August 3), 2009.

## The Study

ILI was defined as fever >38.5°C and cough plus >1 of the following: sore throat, headache, and myalgia. Severe influenza was defined as any case confirmed by reverse transcription PCR in a hospitalized person. The National Ministry of Health provided oseltavimir to every patient >5 years of age who had ILI. Approximately 80% of ILI cases corresponded to pandemic (H1N1) 2009 confirmed by PCR in a pilot study.

All fatal cases included in the analysis occurred in persons whose deaths were directly attributable to influenza. Demographic data, clinical features, concurrent conditions, and number of consultations before hospitalization were recorded (*3*).

Denominators used for determining overall incidence rates and incidence rates by age group were based on the national census of 2002. Denominators used for determining rates for hospitalized case-patients and case-fatality rates (CFRs) were 80% of ILI cases reported to the Ministry of Health during the study. We conducted statistical analysis by using SPSS version 13.0 (SPSS Inc., Chicago, IL, USA) and Epi Info version 6 (Centers for Disease Control and Prevention, Atlanta, GA, USA).

During May 19–August 3, 2009, a total of 342,588 ILI cases were reported. Median age of case-patients was 20.4 years (range <1–109 years). The overall attack rates were 1.2% for pandemic (H1N1) 2009 and 0.4% for persons >60 years of age. During the same period, 651,416 treatments with oseltamivir were reported. Treatment data indicated that the attack rate was 4%.

**Figure 1:**
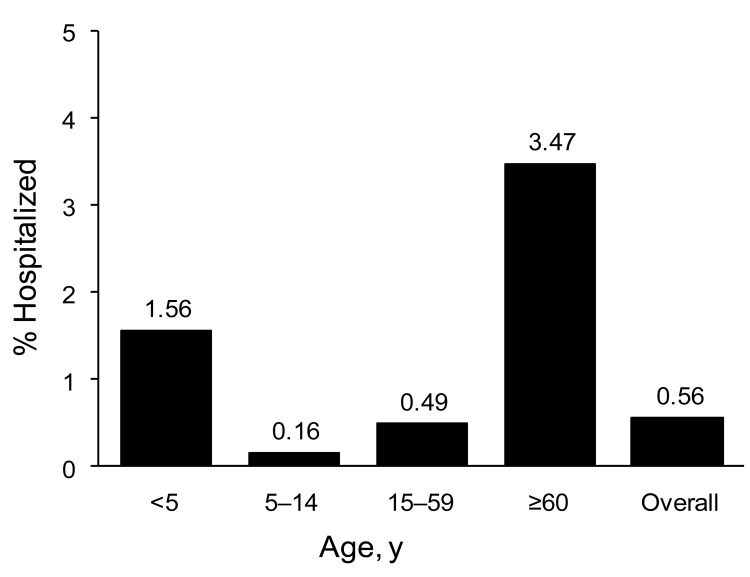
Hospitalization rate for patients with pandemic (H1N1) 2009, by age group among reported case-patients with influenza-like illness, Chile, 2009.

A total of 1,585 persons confirmed to have ILI were hospitalized. Median age was 33 years (range 11 days–94 years), and 52% were women. Overall rate of hospitalization was 9.4/100,000 persons. Case-hospitalization rate was 0.6% (3.5% for persons >60 years of age and 1.6% for children <5 years of age) (Figure 1). Pneumonia was the most common diagnosis at admission (77.3%). Underlying diseases were present in 560 (56.6%) of 989 case-patients, and 217 (22%) had >2 concurrent conditions.

**Figure 2:**
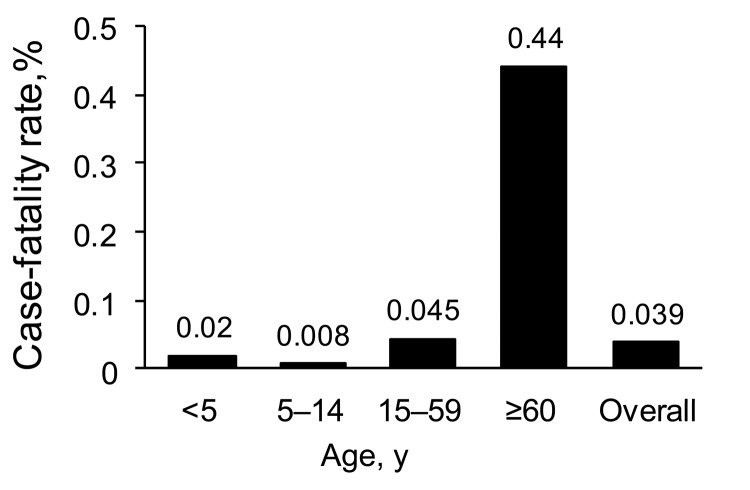
Case-fatality rate for pandemic (H1N1) 2009 by age group among reported case-patients with influenza-like illness, Chile, 2009.

A total of 130 hospitalized patients died (overall mortality rate 0.79/100,000 persons). Among these patients were 117 who died during the study period. Median age was 47 years (range 4 months–89 years) for persons who died and 30 years (range <1–94 years) for persons who survived (p<0.0001). Forty-seven percent of patients who died were >49 years of age. CFR was 0.04%: the highest rate was for patients >60 years of age (0.44%; p<0.0000001), followed by persons 15–59 years of age (0.045%). CFR was 0.02% for children <5 years of age and 0.008% for children 5–14 years of age (Figure 2). Fifty percent of all deaths were caused by severe respiratory failure.

Age was a risk factor for severe influenza and death. Patients >60 years of age and <5 years of age were at a higher risk for severe disease, and patients >60 years of age were at a higher risk for death. At least 1 concurrent condition was identified as a risk factor for death from pandemic (H1N1) 2009. Eighty-four (87.5%) of 96 patients who died and 485 (54.3%) of 893 of patients who survived had an underlying disease (odds ratio 5.89, 95% confidence interval 3.08–11.52; p<0.00001). A concurrent condition was a risk only for persons 15–49 years of age (Table).

**Table:** Risk factors for severe disease or death among patients with pandemic (H1N1) 2009, Chile, 2009*

Risk factor	OR (95% CI)
Severe disease	
Age group, y	
<5	3.66 (3.27–4.11)†
5–14	0.22 (0.18–0.25)
15–59	0.89 (0.81–0.98)
>60	7.94 (6.93–9.10)†
Death	
Age group, y	
<15	0.17 (0.1–0.3)
15–59	1.44 (0.99–2.09)
>60	15.06 (9.94–22.72)†
Concurrent condition	
All patients	5.89 (3.08–11.52)†
Age group, y	
<1	ND (inexact)‡
1–4	3.53 (0.24–101.6)
5–14	ND (inexact)‡
15–49	6.69 (2.4–20.0)†
50–64	2.39 (0.77–8.5)
>64	1.0 (0.18–7.0)

*OR, odds ratio; CI, confidence interval; ND, not defined. †p<0.05. ‡Not significant.

Mean time from onset of symptoms to hospitalization was 4.3 days (range 0–20 days) for persons who died and 3.6 days (range 0–20 days) for persons who survived (p = 0.03). Median time from onset of symptoms to beginning of treatment with oseltamivir was 3.0 days for persons who died and for those who survived.

## Conclusions

The strength of this study was inclusion of nearly all patients with confirmed severe pandemic (H1N1) 2009 in Chile because of timely implementation of a national notification system. The population studied included 97% of hospitalized patients with pandemic (H1N1) 2009.

Differences in attack rate (1.2% by reporting of ILI cases and 4% by amount of oseltamivir provided) might be explained by the fact that not all ILI cases were captured (≈20% were lost because of underreporting) by the notification system and oseltamivir was prescribed to persons with illness not included in the ILI case definition. We demonstrated in a pilot study that ≈60%–70% of oseltamivir used complied with the case definition for ILI.

The first wave of pandemic (H1N1) 2009 in Chile showed the highest incidence rate in children 5–14 years of age; persons ≥60 years of age were least affected (*2*). However, our study identified persons ≥60 years of age as at greatest risk for severe respiratory disease and death, despite the lower incidence rates. During the influenza pandemic, risk for illness in this age group was low but risk for severe disease and death was higher than in the other groups, independent of underlying diseases. This finding differs from results of a study in California, USA, in which persons >60 years of age did not have a high hospitalization rate (*4*). Our finding is consistent with those of a report on seasonal influenza in which risk for severe infection and hospitalization was highest for elderly persons (*5*).

Before April 2009, influenza was rarely reported as the cause of death in Chile. Therefore, only CFRs for severe respiratory infection are available for comparison. The CFRs for patients >65 years during winter 2009 was similar to that during previous winters. We conclude that elderly persons’ risk for a severe outcome during pandemic (H1N1) 2009 did not exceed this risk for a severe outcome during seasonal influenza. Previous reports have documented increased risk for severe outcomes in younger persons during pandemic (H1N1) 2009 and the absence of increased risk for disease severity among elderly persons (*4**,**6**,**7*). On the basis of these findings, some institutions made policy decisions to exclude anyone >65 years of age without concurrent conditions from satisfying recommendations for use of vaccine against pandemic (H1N1) 2009 because of absence of identified increased risk for infection. Our study indicates an age >60 is the greatest risk factor for a severe outcome during pandemic (H1N1) 2009 and seasonal influenza.

Delay in medical care was another risk factor for death in this study. The number of consultations before admission did not differ between the groups, suggesting that patients who died sought medical care later than patients who survived. Thus, timely medical consultation affected patient outcome.

This study indicates that an age ≥60 years was the greatest risk for death associated with pandemic (H1N1) 2009 influenza, similar to that for seasonal influenza. These results can be used for future planning strategies for influenza, strengthening the need for influenza vaccination, opportune medical evaluation, and timely therapy specific for this age group.
